# Resonant Dative Bonds

**DOI:** 10.1002/anie.202515336

**Published:** 2025-10-29

**Authors:** Sebastian Kozuch

**Affiliations:** ^1^ Department of Chemistry Ben‐Gurion University of the Negev Beer‐Sheva 841051 Israel

**Keywords:** Dative bonds, NBO, Pseudo‐carbenes, Resonance, Theoretical molecules

## Abstract

The dative bond is an often underappreciated entity in the theory of chemical interactions, despite its explanatory and predictive potential. In this work, the concept is recovered to computationally design a novel resonant dative bonding pattern in ring molecules, which would be formally considered to be composed of multiple hypervalent and hypovalent atoms. Starting with the (HCPO)_3_ ring arranged as alternating carbons and phosphorus, we propose that the major Lewis structure is composed of P═O double bonds and “semi‐dative‐semi‐covalent” P─C bonds. The three carbons technically are a rare case of σ^0^π^2^ carbenes; however, due to the stability granted by the delocalized, resonant dative bonds, we termed them “*pseudo‐carbenes*”. By experimenting with isoelectronic systems with other elements but with the same bonding pattern, we also observed pseudo‐nitrenes, pseudo‐silylenes, and dicoordinated halogens, among other unusual structures. Surprisingly, the isomeric rings with normal valence and traditional covalent bonds were found to be of much higher energy, suggesting that the resonant dative bonds can potentially be a source of highly stable molecular entities.

## Graphical abstract

Dative bonds are an integral, albeit underappreciated, part of the theory of chemical bonds. In their resonant form they can bring a surprising stability to a novel family of rings with multiple hyper/hypo‐valent elements.

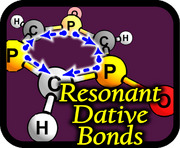

## Introduction

Resonant dative bonds, and the associated semi‐dative‐semi‐covalent bonds that we describe in this article, are not really a novel idea. Indeed, as we describe in this introductory section, many known systems involve these concepts, although we may describe them from a less traditional perspective. The known systems portrayed here as stepping‐stones for further development are the traditional phosphoranes (including the three‐center‐four‐electron bonds), BODIPY with its symmetrical N‐B‐N bonding pattern, and a discussion of methylphosphorane oxide with its relevant Lewis structures. We will later build from these a new (to the best of our knowledge) family of molecules exploiting this bonding pattern, especially ring structures characterized by resonance between their possible Lewis structures, using computational and theoretical means.

### Phosphoranes, an Exemplar of Dative Bonds

**Scheme 1:**
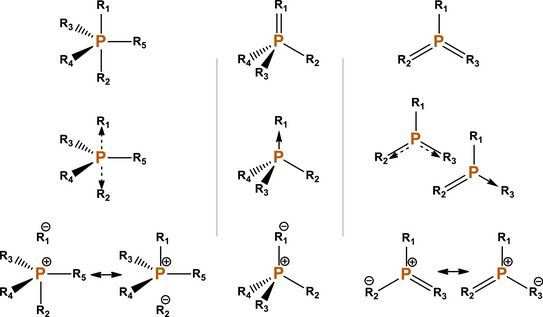
Three types of “hypervalent” phosphoranes. These can be described, from left to right, as σ^5^λ^5^ σ^4^λ^5^, and σ^3^λ^5^, with σ being the coordination number and *λ* the putative valence.^[^ ^16^ ^]^ In the N‐X‐L nomenclature, they are 10‐P‐5, 10‐P‐4, and 10‐P‐3, with N and L being the “formal” electron count and the number of ligands.^[^ ^4^, ^17^ ^]^ In the upper row, the IUPAC recommended skeletal formulae. In the middle row, the representation with dative (arrow) or semi‐dative (dashed arrow) bonds,^[^ ^12^ ^]^ where the octet rule is preserved; the last compound on the right has two main possibilities, one with a pair of π semi‐dative bonds (similar to ozone), and the other with a covalent double bond and a single dative bond, for whom a resonant mixture between these structures is common. In the bottom row, the alternative representation with formal charges (“ylides”), which can be taken as an extreme case of dative bonds where the 1 e^−^ charge transfer is complete; such depiction also fits the octet rule, though it often misrepresents the real electronic distribution^[^ ^12^ ^]^ (see main text and Scheme 3).

Phosphoranes (λ^5^‐phosphanes according to IUPAC,^[^ ^1^ ^]^ see Scheme 1) involve one of the most misunderstood bonding patterns. At first glance, they appear to disrupt the octet rule, having an excessive number of bonds, thus being labeled “hypervalent”.^[^ ^2^, ^3^, ^4^ ^]^ However, many studies revealed that the proper sum of bond orders very rarely deviates from eight, despite the hypercoordination.^[^ ^5^, ^6^, ^7^, ^8^, ^9^, ^10^ ^]^ This indicates that the lines that commonly describe the bonds of these species are misleading and exaggerated, although it is the recommended way to present them (upper row of Scheme 1).^[^ ^11^ ^]^ The simple extra symbol of the dative bond arrow can usually fix the problem (lower row of Scheme 1).^[^ ^12^, ^13^, ^14^, ^15^ ^]^ Sadly, the dative bond and its arrow depiction are neglected topics of chemical education in a large part of the world.^[^ ^12^ ^]^ This is unfortunate, considering that the explanatory and predictive power of the idea in terms of Lewis structures is clear and physically sound within molecular orbital theory.

For example, in describing the prototypical hypervalent Cl_3_P═O, the octet is fulfilled by allowing the electronic lone pair of the Cl_3_P: fragment to be shared with the electronically deficient oxygen atom, which can be represented as a strong Cl_3_P→O charge transfer (this structure is in equilibrium with the one with a P═O double bond and semi‐dative P─Cl bonds). The arrow symbol^[^ ^18^, ^19^ ^]^ represents, in Lewis terms, that the phosphorus does not lose electrons, but the oxygen does gain a pair, therefore fulfilling the octet in all the atoms. In addition, this fits the fact that the computational estimations of the phosphorus‐oxygen interaction do not portray a double bond (it is closer to a 1.5 bond order, but it is very much dependent on the method).

This is a type of oxidation, and therefore the molecule has also been drawn as Cl_3_P^+^–O^–^, a ylide (bottom row in Scheme 1). This nomenclature is also correct in terms of octet electron counting, and for many molecules like phosphoranes, drawing a formal negative charge in the more electronegative atom and a positive charge in the more electropositive often is not far from the expected chemical intuition (for Cl_3_PO, NPA estimates a charge of ‐1.0 in the O, and + 1.7 in the P). Since a dative bond necessarily involves a certain amount of charge transfer, for phosphoranes and other similar molecules^[^ ^20^ ^]^ the ylide depiction reflects, in principle, the same intrinsic physics as the arrow notation. However, despite fitting the octet rule, writing the formal charges suggests an extreme and sometimes misleading case of a complete electron transfer, whereas the real electronic distribution might not fit this description. For example, when depicting carbon monoxide as ^–^C≡O^+^ instead of C≡O,^[^ ^12^ ^]^ a carbanion would be directly attached to an oxonium, implying an unnatural electron density; in contrast, NPA computations show a −0.5 charge in O, with a negligible dipole moment. Considering this, we prefer to use the more general dative bond notation model (although in relevant cases we will also provide the alternative formal charges depiction).

**Scheme 2:**
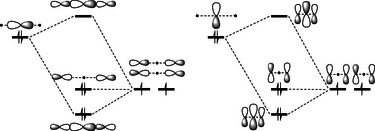
Interaction diagrams for the 3‐center‐4‐electron structures in the σ axis (such as in the axial substituents of PF_5_), or in the π plane (such as in O_3_).

The case of PR_5_, such as in phosphorus pentahalides, involves a 3‐center‐4‐electron bonding between the axial ligands (Scheme 2). In essence, this is also a dative bond, but split between two substituents at ∼180°, each requiring a single electron to complete the octet. Consequently, we called them semi‐dative bonds, and represented them with dative arrows, but with dashed lines as commonly used for half‐bond orders (such as the half π bond in benzene).^[^ ^12^ ^]^ This type of interaction can also occur in the π plane (Scheme 2). For instance, MePO_2_ is a molecule that would fit the rightmost structure of scheme 1, with σ bonds to each ligand, plus the O─P─O 3c‐4e^−^ π interaction. However, a molecule like MeP(CH_2_)O would correspond to a standard P─Me single bond, a P═CH_2_ double bond, and a dative P→O σ bond; this bond combination is central to the present article.

### BODIPY

**Scheme 3:**
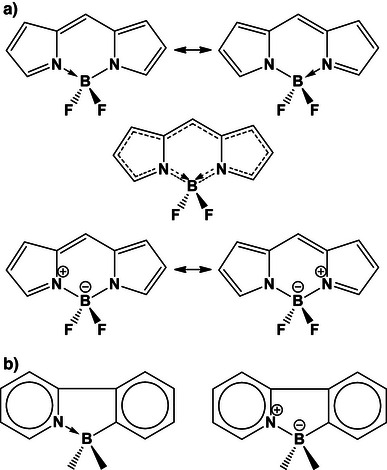
a) BODIPY. In the upper row, the two structures show the resonant π system as well as the covalent and dative B─N σ bonds. In the middle, the mixed structure, highlighting the one‐and‐a‐half C─C and C─N bonds, and the semi‐covalent‐semi‐dative B─N single bonds. At the bottom, the traditional way of depicting BODIPY with formal charges; while this notation fits the octet rule, it tends to be misleading, as the boron is actually positive and the nitrogens negative (with NPA charges of +1.2 for B, +0.15 for BF_2_, and ‐0.5 for each N). b) Two representations of an azaborole, the first with a dative bond, the second with formal charges; with a charge of + 1.1 on B, and −0.4 on N, this second representation is less faithful to the electronic structure of the system.

The bonding pattern that we are looking for has a strong similarity to certain bidentate ligands in metallic complexes (like acetylacetonate or NacNac), and specifically, to N–B←N systems like in BODIPY (Scheme 3).^[^ ^21^ ^]^ In this molecule, the electron‐hungry boron accepts a large amount of charge transfer from the imine, up to the point where both B─N bonds end up being equivalent, resulting in a *C*_2_ *_v_* ground state geometry. This is usually depicted as a resonant structure, with an N─B and an N→B mixture of valence bond structures on each side of the boron (a pair of “
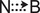
” bonds). These resonant dative bonds can therefore be considered as semi‐covalent‐semi‐dative single bonds. As shown in Scheme 3, it can also be represented with formal charges (it actually is the traditional notation), which fits the octet rule. However, as explained above, this depiction goes against the chemical intuition based on the electronegativity scale and the computed atomic charges, and therefore we favor the arrow‐based dative bond model for these cases.

NRT computations (see below) show a complete octet for all BODIPY atoms (duet for H), with approximately single or 1½ bond orders, as one would expect (B–N = 0.94, B–F = 1.00, C–N = 1.20 or 1.60). Interestingly, both the B─F and B─N bonds show a high amount of ionic character (0.67 and 0.62, respectively, compared to 0.33 and 0.32 covalent bond order). For B─F this is natural, since fluorine is twice as electronegative as boron. But for B─N, the large ionic bond order goes against the electronegativity; this is a sign of dative bonds (the same reason why carbon monoxide has a negligible dipole moment, despite the large electronegativity difference^[^ ^12^ ^]^). Interestingly, NBO identifies the B─N interactions as two equivalent single, covalent σ bonds, instead of one covalent bond and the second a non‐covalent interaction with a large charge transfer (for comparison, the azaborole^[^ ^22^ ^]^ of Scheme 3b has a C─B bond order of 0.95, while the N─B bond order is 0.78, the latter more in line with a slightly weaker dative bond).

### Methylenephosphine Oxide

**Scheme 4:**
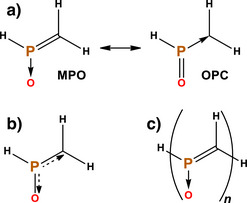
a) Methylenephosphine oxide with its two main resonant structures (**MPO** and **OPC**). Due to the electropositivity of the phosphorus, an ylide representation with a formal positive charge in P and a negative one in O or C is possible in this case. b) A schematic drawing of a possible mixture of the structures, a π 3‐center‐4‐electron bond. c) Studied linear oligomers (with *n* = 1 to 8).

Methylenephosphine oxide is the central motif of this article.^[^ ^23^, ^24^ ^]^ If we accept that this σ^3^λ^5^ moiety is not truly hypervalent, in principle it can be described with two main Lewis resonant structures (Scheme 4a). The first one has a dative bond to the oxo and a double bond to the carbon; we will call this resonant structure **MPO** (acronym of MethylenePhosphine Oxide). The second structure inverts the bond pattern, making a double bond to the oxygen and a dative bond to a carbene, and therefore we will call it **OPC** (for OxoPhosphine Carbene). Following this, we will use the **MPO**/**OPC** acronyms for all the molecules in this article to describe their fundamental Lewis structures, regardless of their chemical formula.

If the **OPC** structure has the greatest weight, then it stands to reason that the molecule would formally be made of a rare σ^0^π^2^ carbene.^[^ ^25^, ^26^ ^]^ In these, the occupied lone pair of the carbene is in an out‐of‐plane p atomic orbital, while the empty orbital will be an sp^2^ hybrid orbital in the plane. Regular carbenes, such as N‐heterocyclic carbenes, usually have the opposite σ^2^π^0^ electronic state (or sometimes a triplet state, such as in methylene^[^ ^27^ ^]^). The otherwise extremely reactive σ^0^π^2^ carbene is obviously highly stabilized by the large charge transfer from the σ dative bonds of the phosphorus to the hybrid atomic orbital in the carbon, in addition to a smaller but significant charge transfer from the p AO of carbon to the P═O π*.

Of note, such stabilization techniques are common in persistent carbenes, although in the opposite direction (N to C charge donation in the π plane, inductive stabilization in the σ plane in NHCs), and with much weaker effects compared to our system. Carbones,^[^ ^28^, ^29^, ^30^ ^]^ and sometimes silylenes,^[^ ^31^, ^32^ ^]^ indeed are stabilized by strong charge transfers from σ electron donor bases (phosphines, carbenes, etc.). The difference between these charge transfer stabilizations in NHC, CAAC, silylenes, and carbones, and the dative bond described in the **OPC** structure might be considered a matter of quantity more than quality.^[^ ^7^ ^]^ But admittedly, the strength of our dative bond is comparable to covalent bonds (see Supporting Information). To be on the safe side in terms of nomenclature, if **OPC** style of bonds occurs, we will consider them *pseudo‐carbenes*.

In this work, we will mix and expand the concepts of resonant dative bonds seen in BODIPY and the equilibrium of Lewis structures in methylenephosphine oxide. While this proposed interaction is not frequent, we suspect that it may appear in more than a limited number of case studies, especially on cyclic structures where formally hypervalent atoms are common.^[^ ^4^, ^33^ ^]^ More specifically, we will try to understand and engineer with computational tools ring structures with alternating hypervalent–hypovalent elements, where the unusual pattern of resonant dative bonds creates particularly stable molecules.

## Theoretical Methods

Preliminary computations showed that several functionals provide the same geometrical and bonding picture (M06‐2X, ωB97XD, PBE, DSD‐PBE‐P86, and even CCSD), already implying that the molecules are probably not of fringe stability. Because this study is of more qualitative nature and less focused on accurate energetics, we simply selected a good functional/basis set combination for main‐group chemistry. Therefore, all computations were performed at the M06‐2X/Def2‐TZVP level^[^ ^34^, ^35^ ^]^ with Gaussian16.^[^ ^36^ ^]^ All energies, including NBO *E*(2) values, are in kJ.mol^−1^.

More important for our purpose is the selection of the bonding analysis techniques. For this, the most useful family of methods to examine strong bonds in terms of molecular orbital theory is the natural bond order analysis (NBO), of which we are using version seven.^[^ ^37^ ^]^ Beyond the usual information provided by NBO, we also employed natural resonance theory (NRT).^[^ ^38^ ^]^ NRT recovers molecular information in the emblematic chemical language of Lewis electronic structures and their resonance weights, but based on strict quantum methods instead of using heuristic logical rules. This is a practical method for semiquantitative analysis of the resonance weights of Lewis structures, although it is not without controversies.^[^ ^39^, ^40^, ^41^ ^]^ Unfortunately, when molecules grow in complexity and size, the number of resonant structures also increases, including “intruder states” (that is, lower weight states with structures not relevant for the discussion at hand). Although the use of NRT weights is a powerful method, for our project this issue provided fluctuating and unreliable results that could not aid in the rationalization of the trends (fine‐tuning the NRT keywords and thresholds sometimes helped, but not always, see Supporting Information). We could only obtain meaningful NRT information for the methylenephosphine oxide monomer (see below). Therefore, except for some simple cases, we prefer to use other gauges, including NRT bond orders^[^ ^42^ ^]^ (the integer bonding of each structure multiplied by their weights, which we found to be a much more robust measure than the resonance weights themselves, and it seems to work better specifically for dative bonds compared to Wiberg on the basis of NAOs) and the valency index, plus other regular NBO measures such as bond occupation and perturbative interaction energies.

To address the NRT issue on large or complex systems, we devised a simple method to estimate the weight of selected resonant structures. This technique is based on the comparison of the *E*(2) perturbative interaction energies between different NBO structures (selected with the $CHOOSE keyword).^[^ ^37^ ^]^ In essence, if an NBO set that can be identified with a Lewis structure has negligible *E*(2) values, then the involved NBO orbitals are a good description of the “real” orbitals. However, if there is a large donor‐acceptor stabilization energy between key orbitals, then the NBO set is not an accurate picture, since it lacks the delocalization correction. Usually, similar information can be obtained from the NBO partial orbital occupation. Because of this, second‐order NBO perturbational analysis is useful to understand chemical concepts such as hyperconjugation or charge transfer when working within Lewis structures and localized molecular orbital models. It stands to reason that when comparing two resonant structures, the one with a larger *E*(2) value will deviate more from the real electronic state. Therefore, the ratio between the *E*(2) numbers of the two sets should be a good gauge of the weight of the structures. We found that NRT weights and these “NBO ratio” values correlate, making the latter a good and simple proxy for estimating the weights of selected resonant structures when NRT tendencies appear technically erratic. In addition, it matches the result of a comparative test against valence bond theory.^[^ ^43^ ^]^ The details of the tests and of the method appear in the Supporting Information. We expect the NBO ratio method to provide only semi‐quantitative values, as it is biased in favor of only two possible Lewis structures, and while it was carefully tested, it was only applied on a small number of model reactions. Although the values obtained for specific systems should be taken with caution, we believe that tendencies obtained with the NBO ratio method remain reliable.

## Results and Discussion

We will start by analyzing the electronic structure of methylenephosphine oxide, trying to answer which of the two structures in Scheme 4a is more accurate. Natural resonance theory was built to deal with these questions, putting a weight on each possible structure (although, as said above, it gets challenging with more complex systems). Interestingly, the answer was an almost identical mixture of **MPO** and **OPC**. The latter has a weight of 50.7%, while the former involves two similar structures of 30.7% and 18.6% (summed to 49.3%), depending on the position of the oxygen lone pairs (there is no structure with both P═O and P═C double bonds in the NRT computation). Consistently, the bond orders are 1.49 and 1.51 for P─O and P─C, respectively. This suggests that the 3‐center‐4‐electrons pattern in the π plane (Scheme 4b) is formally correct within the NRT technique.

**Figure 1:**
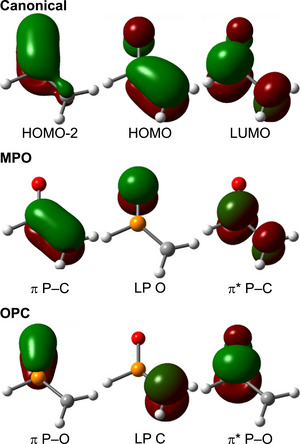
Canonical and NBO orbital sets in the π system of methylenephosphine oxide. The **MPO** NBO set is based on a P═C double and P─O single bond (the default NBO structure), while the second NBO structure swaps the orbital order (P─C, P═O), forming the **OPC** electronic structure. The NBO set with larger charge transfer from the lone pair to the π* antibonding orbital indicates a more contaminated electronic structure, and therefore with less resonant weight.

In Figure 1 we depict the orbitals of the π system of methylenephosphine oxide in the canonical as well as in two NBO sets, corresponding to the **MPO** and **OPC** resonant structures. In the canonical MOs, we see one P─O bond (HOMO‐2), and another P─C bond which is also slightly P–O antibonding. These MOs match the particle in a box (skewed by the electronegativity difference), and the 3‐center‐4‐electrons (Scheme 2) models. In NBO, we have one set with a P─C π bond and a lone pair in the oxygen (**MPO**, the default configuration), and another set with a P─O π bond and a lone pair in the carbon (**OPC**). In both cases the lone pairs have very strong interactions with the π* at the other atoms, explaining the resonance of scheme 4a. In the **MPO** set, the LP(O)→π*(P─C) donor‐acceptor stabilization is 259 kJ.mol^−1^, while for the **OPC** the LP(C)→π*(P─O) gives a higher value of 545 kJ.mol^−1^. With an **MPO**:**OPC** NBO ratio of 68:32 (see Supporting Information), this technique implies that the former [the H_2_C═P(H)→O depiction] is closer to the real state. However, if we trust the almost 50:50 NRT weights, we can take this NBO ratio as a reference value, and argue that any molecule with a smaller NBO ratio will show preference for an **OPC** structure (as explained in the SI, there is a strong correlation between the two methods, but the intercept does not need to be zero).

### Linear Methylenephosphine Oxide Oligomers

**Figure 2:**
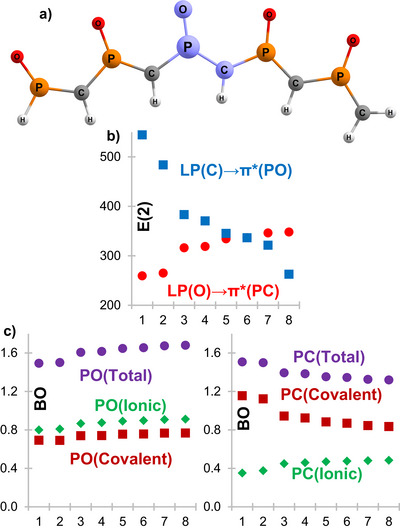
a) Example of a linear oligomeric methylenephosphine oxide with *n* = 5; in purple the central atoms from where the NBO and NRT values were obtained. b) Donor‐acceptor stabilization energies for the two sets, in kJ.mol^−1^ (**MPO** in red, **OPC** in blue). c) NRT bond orders between the central atoms, including covalent and ionic components.

The physical expansion of the molecular system can severely affect the resonant forms. Therefore, we linearly expanded the methylenephosphine oxide motif as shown in Scheme 4c and Figure 2a, using a *trans* planar conformation to minimize other effects (a *cis* conformation is slightly more stable due to intramolecular H‐bonds, see Supporting Information). We compared the properties of the systems with *n* = 1 to 8, only considering the resonances of the central unit to reduce border effects. Similarly to the monomer, we carried out NBO and NRT bond order analysis to detect the nature of this middle section in terms of **MPO** or **OPC** character (see Figure 2).

Although there is no complete linearity, the trends are clear. The charge transfer stabilization energy (Figure 2b) and the bond orders (Figure 2c) show the strengthening of the P─O and the reduction of the P─C double bond character, with the concomitant increase of the **OPC** at the cost of a decrease in the **MPO** weight (the **MPO**:**OPC** NBO E(2) ratios go from 68:32 for *n* = 1, to 43:57 for *n* = 8). All this is a sign that small chains can be better described as R_2_C═P(R)→O, whereas large chains are more **OPC** like, that is O═P(R)→CR_2_. If we take the NRT weights of the monomer as a reference, then all the oligomers are mostly pseudo‐carbenes in the center of the molecule.

An interesting observation is that the lowering of the P─C bond order comes from its covalent nature, and it goes against the heightened ionic character of the interaction (right side of Figure 2c). As explained above, a larger ionic/covalent ratio is a characteristic of dative bonds, supporting the hypothesis of the formation of a phosphine‐stabilized pseudo‐carbene (or at least, a resonant structure with carbene‐like character). The thermodynamical molecular stability also seems to improve with the length (see Supporting Information), which might be connected with the trend of the electronic structure.

### Cyclic Methylenephosphine Oxide Oligomers

**Scheme 5:**
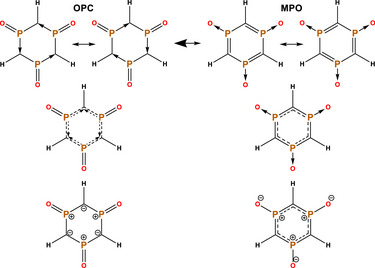
Top: **OPC** and **MPO**, the two main resonant structures of triphosphinine trioxide, each one with two underlying resonances. Middle: The semi‐dative‐semi‐covalent bonding for **OPC** and the aromatic structure for **MPO**, with dotted lines indicating one e^−^ interactions. Bottom: The alternative ylide depiction, which is possible due to the electropositive nature of the phosphori.

Since all the previous discussion involved only the central O─P─C unit of the linear chains, the natural following step would be to examine the situation where there are no border effects at all, namely in rings. For that, we designed a triphosphinine trioxide, (HCPO)_3_, a three‐unit cyclic species which may be favored by the typical stability of six‐membered rings (Scheme 5). The resulting LP(O)→π*(P‐C) and LP(C)→π*(P─O) E(2) perturbational energies are 336 and 284 kJ.mol^−1^, respectively, with an **MPO**:**OPC** NBO ratio of 46:54. This suggests that once again the σ^0^π^2^ pseudo‐carbene seems to be the structure with larger weight. The NRT bond orders for P─O and P─C are 1.63 and 1.12, also showing the greater preference for the **OPC** resonant structure.

We also computed the NICS(1)_ZZ_ value^[^ ^44^ ^]^ of this ring as a qualitative method to test if the system adheres more to **MPO** or **OPC** electronic characteristics. This measure is obtained by computing the NMR tensor, and extracting the value perpendicular to the plane of a ghost atom centered at 1 Å over the ring. Large negative values signal a strong deshielding caused by the diatropic current of the aromatic system (the ZZ component at 1 Å improves the individualization of the π component, minimizing the interference from the σ system). For example, the archetypal benzene has a NICS(1)_ZZ_ value of −30.1, while for 1,3,5‐triphosphinine (the aromatic C_3_H_3_P_3_ heterocycle) it is −23.4. If **MPO** was the primary electronic structure of (HCPO)_3_, we would expect it to provide similar NICS(1)_ZZ_ results as the latter, caused by the fully resonant P─C π system between three π bonds, matching the 4n +2 Hückel rule. However, this ring provides a value of −5.0, essentially a negligible aromaticity, more in line with an **OPC** structure. Of note, if **MPO** state would be the major state, it would have an extra aromaticity stabilization. In contrast, **OPC** has twelve electrons in the plane of the ring for six σ bonds, cancelling the possibility of σ aromaticity. In other words, the system is stable despite the lack of aromaticity.

In the resulting *D*_3h_ symmetry there is no difference between the P─C bonds. Therefore, we cannot pinpoint one dative and one covalent bond emerging from each phosphorous, but rather a mixture of them (middle left of Scheme 5). These are multiple resonant dative/covalent bonds, akin to BODIPY (Scheme 3a).

The arrangement may formally be considered to be a tri‐carbene (or a tri‐pseudo‐carbene), but with delocalized bonds. The system also seems to be stable in kinetic and thermodynamic terms (see Supporting Information), and therefore we believe it to be, on paper, synthesizable. To the best of our knowledge, this (HCPO)_3_ molecule and its resonant dative bonding pattern are novel additions to the chemical space.

We also considered ring systems of other sizes. Larger rings are unstable to intramolecular bonding, producing ladderane‐style geometries of more traditional valency (tetracoordinated carbon, pentacoordinated phosphorus, see geometries in the Supporting Information), and, therefore, we did not pursue them. The (HCPO)_2_ has an even higher P─O bond order of 1.76, and a lower C─P order of 1.04, with a 45:55 NBO ratio. All this points to a slightly more marked σ^0^π^2^ carbene resonant structure. However, this square molecule is less stable than the six‐membered ring. To obtain a final test of stability of the parent ring, we also computed the possible ladderane and prismane isomers of (HCPO)_3_; they are minima, but they have a significantly higher energy compared to the ring (106 and 140 kJ.mol^−1^, respectively; see geometries in the Supporting Information).

**Scheme 6:**
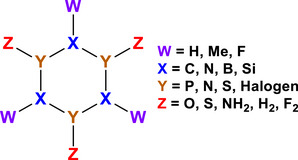
Letter coding for the (WXYZ)_3_ six‐member ring molecules based on the parent (HCPO)_3_ system.

**Figure 3:**
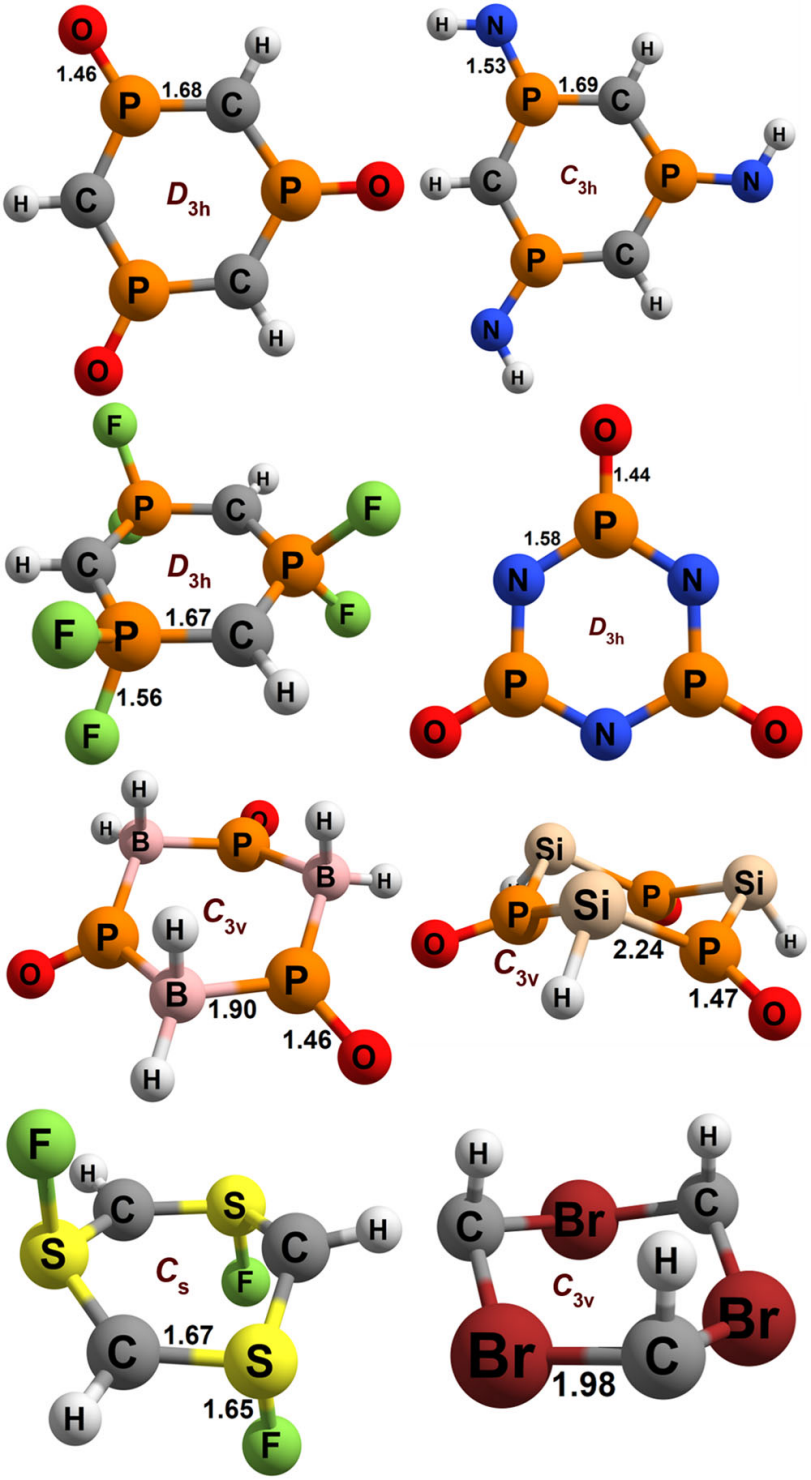
Geometries of selected six‐member rings, including their symmetries and bond lengths in Å.

For the sake of completeness, we computed several other isoelectronic cyclic systems with the same structure, in order to understand whether the previous observations are generalizable. In other words, to check if the “resonant dative bond” can be a common chemical pattern. For that, we symmetrically substituted all the atoms of the (HCPO)_3_ ring in various combinations, which we will denote as (*WXYZ*)_3_, as seen in Scheme 6. We show below the main computational observations, leaving the complete tables to the Supporting Information. In Figure 3 we present the geometries of selected examples.

### Substituents at the Carbons

The most obvious and simple substitution would be to swap the hydrogens bonded to the carbon with an electron withdrawing or donating group, that is fluoride and methyl. The differences with the parent molecule were not significant, with the NBO **MPO**:**OPC** ratios being 50:50 and 46:54 for Me and F, respectively, indicating that more electronegative substituents slightly favor the carbene electronic structure, although the π donation from F to C can also be a factor.

### Substituents at the Phosphorous

More interesting effects can be obtained by carrying out substitutions on the phosphorous. With *Z* = S, the geometry is similar to the parent (HCPO)_3_. However, the NBO **MPO**:**OPC** ratio falls to 54:46, showing an electronic structure significantly less carbene‐like, and more **MPO**, at least according to this measure. But when setting *Z* = NH, we observe an NBO ratio of 39:61, an enhancement of the **OPC** π carbene structure.

Even more extreme results are obtained by splitting the P─*Z* bond in two by forming PH_2_ or PF_2_ substructures (see Figure 3). This foments a tetracoordinated and formally tetravalent phosphorous, with an undoubtedly **OPC** resonant pseudo‐carbene electronic structure. The σ bonds of P─F or P─H impede the dative bond that was possible for the P─O interaction in the **MPO** structure, which hinders in turn the P═C double bonds. In Lewis and Pauling terms, the electronic structures and resonances of **MPO** are more problematic compared to the viable structures brought by the double P═O bonds. Consequently, we obtained an NBO ratio of 3:97 for *Z* = H_2_, whereas for *Z* = F_2_ all the attempts to generate an **MPO** structure failed. Moreover, the NICS(1)_ZZ_ value of the ring is ‐3.7, showing its negligible aromaticity that would have appeared with an **MPO** state. These are patent cases of resonant dative bonds with formally hypervalent λ^4^ phosphorous, and hypovalent λ^3^ carbons (or, alternatively, a triple ylide system if the formal charge notation is preferred).

It is possible to generate a ring isomer of (HCPF_2_)_3_ with normal valency (tricoordination on P, tetracoordination on C) by moving one fluoride from each phosphorous to each carbon. This molecule can be used as a strict stability test for the herein described resonant dative bonds against an “acceptable” system with traditional coordination. Unexpectedly, the most stable conformation we found of this isomer (see Supporting Information) is 445 kJ.mol^−1^ higher than the formally hypervalent P – hypovalent C molecule. Such a large value attests to the surprising strength of the resonant dative bond. The above‐cited virtually insignificant NICS value also indicates that π aromaticity is not playing a role in the stability of these molecules.

### Substitutes for the Carbons

If we speak of isoelectronic molecules, we can also substitute the atoms of the ring themselves, for example, by swapping the HC units with nitrogens, forming the (NPO)_3_ ring. With this change, the **MPO**:**OPC** ratio results 37:63, indicating a larger **OPC** character than in the parent system. In other words, (NPO)_3_ seems to be mostly O═P(R)→N substructures. More electronegative species are better dative bond electron acceptors, which justifies the stability of the **OPC** electronic structure. This can be understood if we imagine an extreme case of full electron donation, creating an ylide: R_3_P^+^–^−^NR_2_ is a more reasonable structure than R_3_P^+^–^−^CR_3_ (although, as seen above, this explanation is not universal, since changing the *Z* group to S or NH had the opposite effect). The P─*X* bond order is slightly lower (1.02 for *X* = N versus 1.12 for *X* = C), and the P─O bond order is higher (1.71 versus 1.63). In other words, (NPO)_3_ can be considered as a multiple σ^0^π^2^ *pseudo‐nitrene*. This entity is bound, again, by strong, resonant, σ dative bonds.

Another option is to replace the CH fragments with BH_2_, forming (H_2_BPO)_3_. By doing so, the weight of the **OPC** electronic structure is maximized, with NRT bond orders of 2.02 and 0.94 for the P─O and P─B bonds, respectively. The molecule does not even permit a clear NBO structure of the **MPO** type, and in the **OPC** structure there is no hint of a P═B double bond (which, again, might fit into a triple ^+^P─B^−^ ylide notation). This is not surprising, since the BR_3_ group naturally is an excellent σ acceptor, but a very bad π bond generator. This lack of π bonds also hinders the *D*_3h_ geometry that appeared in all previous cases, favoring a *C*_3v_ symmetry (Figure 3). The stability of the resultant resonant dative motif is born from the same effect seen in BODIPY.

**Figure 4:**
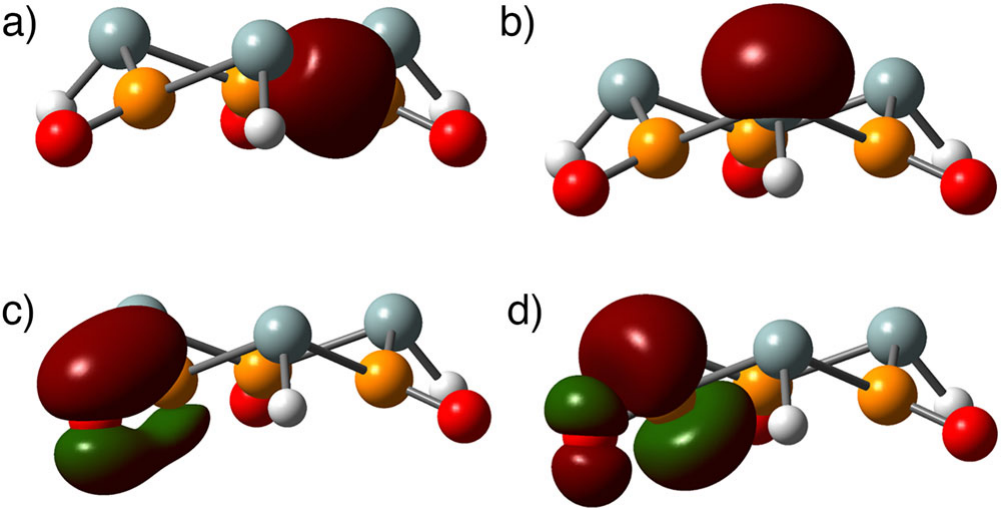
Selected NBOs of (HSiPO)_3_. a) Semi‐dative‐semi‐covalent σ P‐Si bond. b) sp^3^ lone pair on the silicon. c) π P─O bond. d) Virtual π* P─O orbital.

A more consequential effect occurred when setting *X* = Si. Elements of the third row of the periodic table are reluctant to form π interactions, which in this case resulted in a complete change in symmetry and bonding pattern compared to the carbon counterpart. Instead of creating σ resonant dative bonds in the plane and creating lone pairs in the p atomic orbital of atom *X* (like in the carbenes and nitrenes), for (HSiPO)_3_ the phosphorus binds directly to the p AO in silicon. As a result, there is a distortion of the planar symmetry, generating a pyramidal geometry at the silicon centers (see Figures 3 and 4). This leads the silylene lone pair to appear as an sp^3^ lobe (with an E(2) perturbational energy of only 34 kJ.mol^−1^ to the π* of the P═O bond). In other words, (HSiPO)_3_ is a triple σ^2^π^0^ *pseudo‐silylene*, in contrast to the σ^0^π^2^ carbenes and nitrenes described above. Such a kind of silylene stabilization is not unheard of,^[^ ^31^, ^32^ ^]^ and it is the same effect that allows the existence of carbones.^[^ ^28^, ^29^, ^30^ ^]^

### Substitutes for the Phosphori

The final possibility is to switch the phosphorus for other isoelectronic groups, with the first natural choice being nitrogen. Consistent with the previously explained point, (HCNO)_3_, with the more electronegative *Y* atom, is a worse candidate to provide a dative bond to the carbon. Its NBO ratio is 86:14, with a P─O bond order of 1.33 and 1.21 for N─C, that is, a major **MPO** electronic structure. If we also substitute the HC fragment with a nitrogen, forming the (NNO)_3_ compound, then the NBO ratio results 36:64 with N─O and N─N bond orders of 1.59 and 1.07, now more consistent with an **OPC** pseudo‐nitrene.

**Figure 5:**
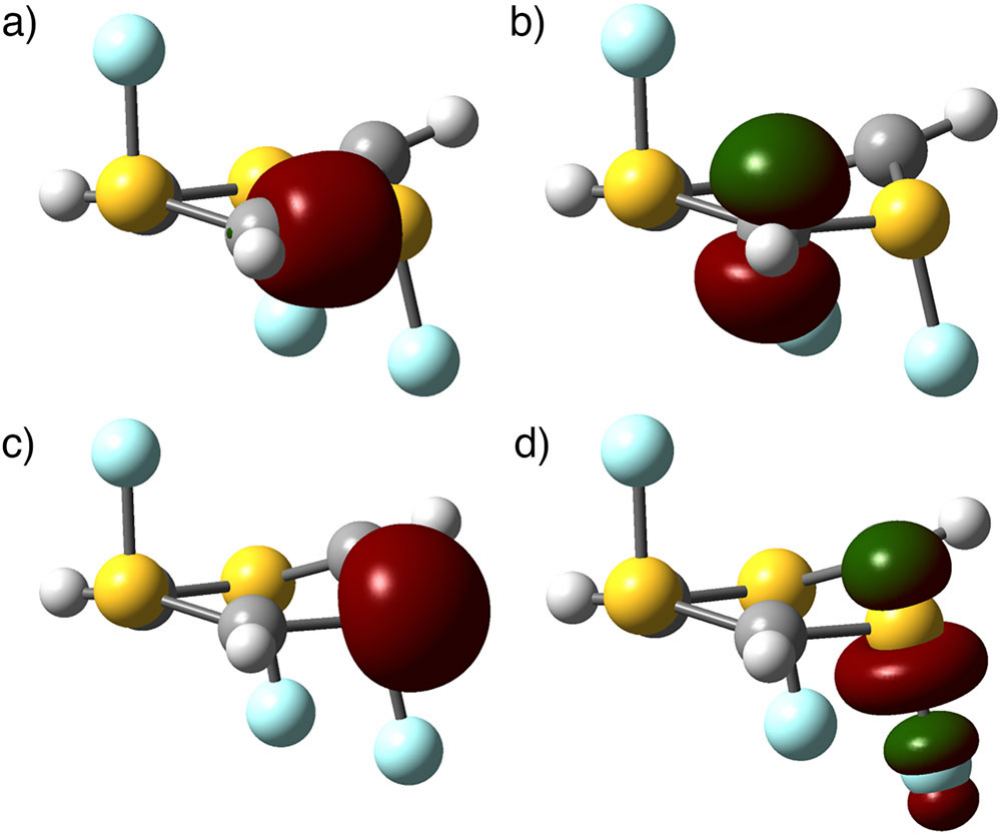
Selected NBOs of (HCSF)_3_. a) Semi‐resonant‐semi‐covalent σ S─C bond. b) p lone pair of the σ^0^π^2^ pseudo‐carbene. c) Sulfur's lone pair. d) Virtual S─F σ*, which interacts with the carbon p lone pair.

When replacing the whole P═O groups with the isoelectronic but coordinately dissimilar S‐F units, we obtain a qualitative geometrical change, turning into a *C*_s_ symmetry. As shown in Figures 3 and 5, the distortion is not occurring at the *X* atom like in (HSiPO)_3_, but at the sulfur, with the fluorides pointing at almost a right angle to the plane. This allows an optimal superposition between the carbon p AO and the S─F σ* [E(2) = 210 kJ.mol^−1^], as depicted in Figure 5. An alternative NBO structure more similar to the **MPO** state with a double C═S bond would require breaking the S─F bond and forming an S^+^F^−^ state, which has enormous LP(F)→π*(C─S) and LP(F)→σ*(C─S) charge transfers of over 1 MJ.mol^−1^, indicating a negligible **MPO** and a major **OPC** electronic structure. The geometry bears some similarities with (HSiPO)_3_, but in this case, it is a resonant dative σ^0^π^2^ pseudo‐carbene.

**Figure 6:**
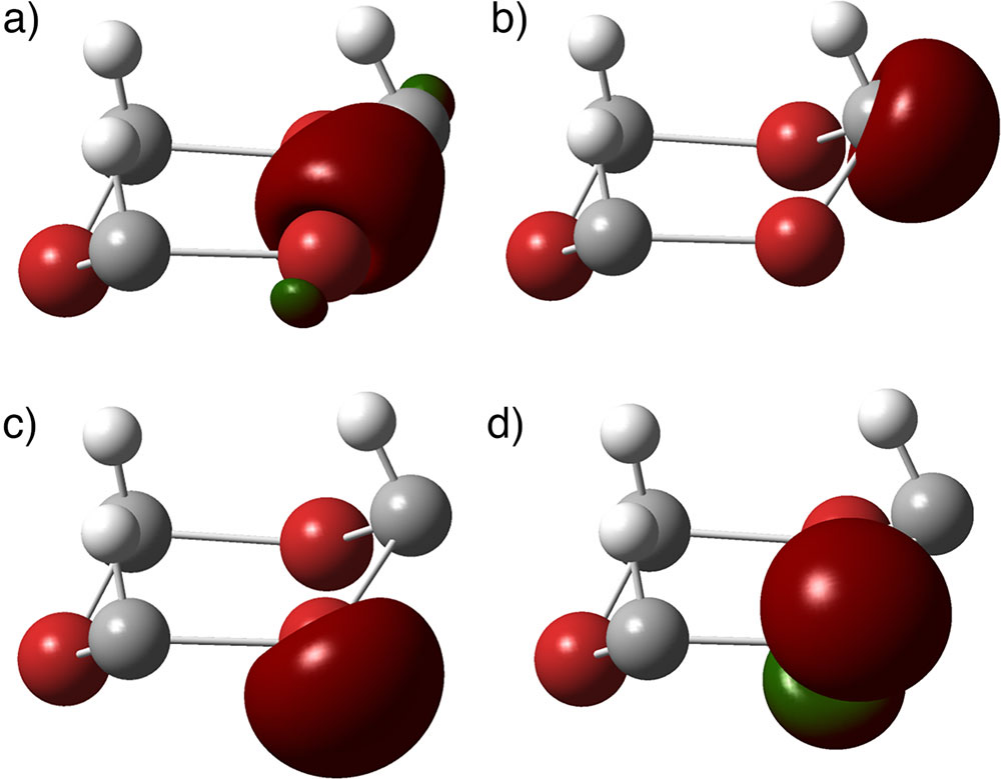
Selected NBOs of (HCBr)_3_. a) Semi‐resonant‐semi‐covalent σ C─Br bond. b) sp^3^ lone pair on carbon. c) and d) Lone pairs on the bromine.

The final family of molecules considered here contains dicoordinated halogens replacing the P═O groups. The stability of these systems requires a careful examination. Due to the strong π charge transfer from the fluorides, (HCF)_3_ breaks apart to three HCF carbenes in a barrierless process. However, with *Y* = Cl, Br, or I, the six‐member rings are favored by 143, 188, and 262 kJ.mol^−1^, respectively. In all of these, we observe that the symmetry falls to *C*_3v_ (Figures 3 and 6), with resonant dative bonds resembling the above described (HSiPO)_3_. Heavier halogens are not able to fully interact with the carbons through π bonds, and therefore they create σ resonant dative bonds to hybrid orbitals at the carbons. All the NBO perturbational interactions are negligible, and we could not find any other meaningful NBO set beyond the default, which encompasses two lone pairs in the halogens, and one sp^3^ lone pair in the carbon (Figure 6). In other words, all these hypervalent halogen–hypovalent carbon molecules can be considered as resonant dative stabilized σ^2^π^0^ pseudo‐carbenes. Correspondingly, the halogen‐carbon NRT bond orders are close to one. Halogenated molecules with nitrogen instead of CH [that is, (NF)_3_ to (NI)_3_] are also possible compounds with chair *C*_3v_ geometries and very similar characteristics, with the only difference that the fluorinated ring is kinetically stable. In the same line, the (H_2_BF)_3_ ring is stable, with the same resonant dative bond as in BODIPY. All these structures with multiple hypervalent halogens are untenable chemical entities if we overlook the existence of the dative bond^[^ ^12^ ^]^ (or, alternatively, a triple carbanion‐halonium ring in a multi‐ylide system).

## Conclusion

Dative bonds^[^ ^12^, ^13^, ^14^, ^15^ ^]^ is an often overlooked^[^ ^12^ ^]^ but an extremely useful addition to the basic toolbox of Lewis structures and molecular orbital theory. It provides a rich chemistry with exceptional explanatory capabilities, especially for putative hyper‐ or hypovalent atoms, where simple Lewis structures lacking the “dative arrow” tend to provide unsatisfying depictions. Moreover, it has remarkable predictive power for the conception of novel molecules and bonding patterns.

In this paper, we computationally designed a new family of molecules based on what we call *resonant dative bonds*. On the basis of known systems (phosphoranes, BODIPY, and methylenephosphine oxide), a ring system stabilized by these bonds was theoretically assembled. This ring is composed of P═O units, bound through a covalent P─C bond on one side, and on the other generating a dative bond to a formally σ^0^π^2^ carbene (termed here pseudo‐carbene). Three of these units create a stable six‐membered ring of three formally “hypervalent” P and three “hypovalent” C. In such a symmetric structure, the covalent and the dative bonds are mixed and delocalized, creating resonant semi‐dative‐semi‐covalent interactions.

Based on this motif, by altering and isoelectronically tweaking the atoms of this cyclic (HCPO)_3_, we also developed several molecular entities with unique electronic states. This includes multiple pseudo‐nitrenes or pseudo‐silylenes, switching between σ^2^π^0^ and σ^0^π^2^ pseudo‐carbenes, or having dicoordinated halogens, among other effects. Noteworthy, the “hypervalent‐hypovalent” version of (HCPF_2_)_3_ is considerably more stable than the acceptable octet and Lewis isomeric ring with tetracoordinated carbons and tricoordinated phosphorus. This suggests that resonant dative bonds might be more than an academic curiosity, potentially generating strong bonding patterns of high stability.

## Supporting Information

XYZ geometries, stability analysis, full NBO tables, and details of the NBO ratio analysis. The author has cited additional references within the Supporting Information.^[^ ^45^, ^46^, ^47^, ^48^ ^]^

## Conflict of Interests

The author declares no conflict of interest.

## Supporting information



Supporting Information

## Data Availability

The data that support the findings of this study are available in the Supporting Information of this article.
