# Supplementary material for: Resonant Dative Bonds
Source: Angew Chem Int Ed Engl. 2025 Oct 29;64(52):e15336. doi: 10.1002/anie.202515336 (PMC12723455; doi:10.1002/anie.202515336)
Supplement: Supplementary file 1 — Supporting Information [file ANIE-64-e15336-s001.pdf]

Supporting Information for

Resonant Dative Bonds

Sebastian Kozuch

Department of Chemistry, Ben-Gurion University of the Negev, Beer-Sheva 841051, Israel

**NBO Ratio**	**S2**
**NBO and NRT Tables**	**S4**
**Stability Analysis**	**S6**
**XYZ Geometries**	**S7**

NBO Ratio

As explained in the main text, the E(2) perturbational interaction energy between NBOs can be used as a measure of the dissimilarity between the localized orbitals and the “real” orbitals. As such, when comparing two NBO resonant structures (selected with the $CHOOSE keyword), if there is a clear perturbational term describing the particular resonance interaction of interest on each structure, then the ratio between them should indicate the weight of the respective Lewis structure. If these NBO ratios provide a correlation with the NRT weights, then it can be used as a simple and reasonable way to semiquantitatively gauge the influence of selected Lewis structures. This is useful when NRT generates many irrelevant resonances of low weight for the problem at hand (what we termed “intruder states”). These states can be of a different symmetry than the one we are looking for (such as σ resonances when looking at the π system), or electronic structures that look chemically odd.

We took the peptide bond of formamide, HC(=O)-NH_2, as an archetypal case of resonance to analyze the issue. Classic textbooks speak of two main Lewis structures, **1** and **2** in Textbox S1. However, a standard NRT computation already provides seven structures, three of them numerically negligible, and two more chemically irrelevant according to our “chemical intuition”, as they break the σ bonds (**3** and **4** in Textbox S1). One way to filter out the intruder states is to adjust the energy threshold with the NRTE2 keyword to select states (NRTE2=5 kept four structures, NRTE2=50 kept two). This seemed to be very useful, but the trick was intractable with the multiple resonances of systems like $(HCPO)_3$. Nevertheless, it worked well for this test.

We used formamide and substituted the oxo group with S, Se, Te, and NH. For each, by selecting a suitable NRTE2 value, we could compute the relative NRT weights solely between the two relevant states (**1** and **2**). The NBO ratios were obtained from the E(2) values of the LP(X)→BD*(C-N) and LP(N)→BD*(C-X) perturbational interactions [$E(2)_1$ and $E(2)_2$, respectively], and simply calculated as

$$\text{NBO 1} = \frac{E(2)_2}{E(2)_1 + E(2)_2} \times 100 \qquad \text{NBO 2} = \frac{E(2)_1}{E(2)_1 + E(2)_2} \times 100$$

Textbox S1. Formamide, its four leading Lewis structures, the connectivity of the most relevant one, the resonance weights, and the bond orders, as appears in the NRT output.

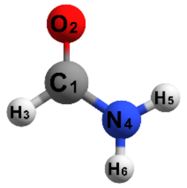

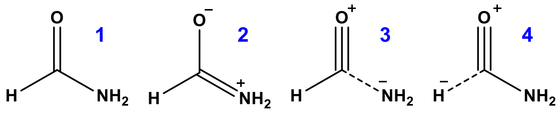


```
TOPO matrix for the leading resonance structure:

    Atom  1   2   3   4   5   6
    ---- --- --- --- --- --- ---
  1.  C   0   2   1   1   0   0
  2.  O   2   2   0   0   0   0
  3.  H   1   0   0   0   0   0
  4.  N   1   0   0   1   1   1
  5.  H   0   0   0   1   0   0
  6.  H   0   0   0   1   0   0

          Resonance
    RS    Weight(%)     Added(Removed)
 ---------------------------------------
    1       46.15
    2       32.12     (C1-O2),C1-N4,O2,(N4)
    3       11.97      C1-O2,(C1-N4),(O2),N4
    4        8.46      C1-O2,(C1-H3),(O2),H3
    5        0.69     (C1-O2),C1-N4,(N4-H6),H6
    6        0.40     (C1-H3),C1-N4,(N4-H5),H5
    7        0.19     (C1-O2),C1-N4,(N4-H6),O2
  others     0.00
 ---------------------------------------
           100.00    * Total *

Natural Bond Order:
C-O 1.8744
C-N 1.2143
C-H 0.9113
```

The relationship between the NRT and NBO ratios can be observed in Fig. S1 and Table T1. There is an almost perfect correlation with the chalcogen substituents, while the NH, providing some different chemical effects, slightly deviates. Still, it is clear that the NBO ratio can produce relatively straightforward estimations of the resonance weights when NRT does not provide a clean picture. This works especially well when one NRT reference is used to compare with (like methylenephosphine oxide in our project), since the intercept of the linear trend is not zero.

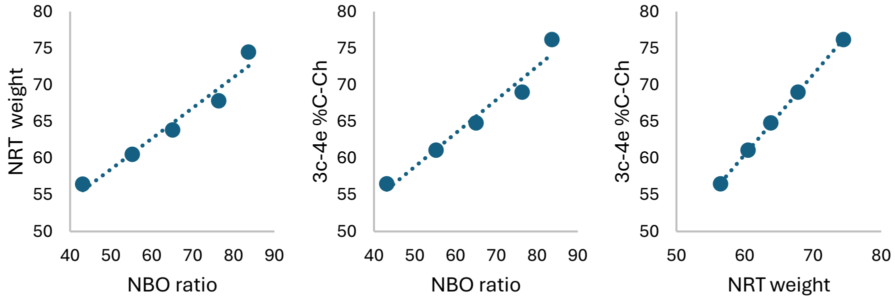

X	NRT	NBO	3C-4e$^-$
NH	74	84	76
O	68	76	69
S	64	65	65
Se	61	55	61
Te	56	43	57

Figure S1 and Table S1. For HC(=X)-NH2, relationship between the percentage of the resonant structure **1** for

- NRT weight of **1** (in comparison with **2**)
- NBO ratio of **1**
- C-X weight in the 3-center-4-electron NBO estimation of **2**.

We also observed that the %A-B character of the resonant hybrid in the 3-center-4-electron "hyperbond" NBO search essentially gives the same information as the NRT weights (Fig. S1 and Table T1). However, this information is only given when the program finds such a pattern, which seldom occurs. For instance, we could extract the information when studying structure **2**, but for HC(=O)-NH_2 and HC(=NH)-NH_2 in the default Lewis structure **1** it does not appear. Still, if the 3-C-4-e$^-$ analysis appears in a particular set of reactions of interest, the results can be used instead of the NRT computation.

We finally considered the Chirgwin-Coulson valence bond weights computed with BOVB with XMVB software for the two first resonant structures of formamide, obtaining a 77:23 relation between them. This is essentially the same as the results with the NBO ratio method, further justifying our results.

NBO and NRT Tables

For simplicity, NRT analysis for the linear structures was performed by selecting only one set of O-P-C atoms. A complete NRT study including all the atoms does not provide a significant difference in terms of bond orders or valencies, but blurs and complicates the results by adding many more intruder states, especially for long chains. For cyclic molecules, we used zwitterionic structures (with the *nrtchg* option) to allow for symmetrical resonances, which do not naturally appear with the default options.

Table S2. NBO Occupation values, perturbational energies in kJ.mol^{-1}, and NBO ratios.

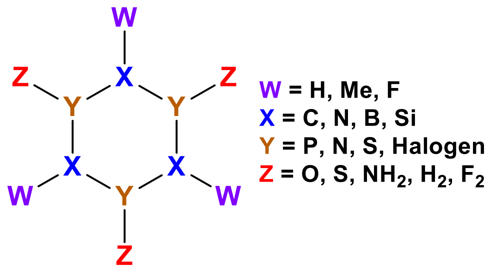

		MPO			OPC			NBO Ratio
	Symm	LP Z	BD* XY	E(2)	LP X	BD* YZ	E(2)	MPO:OPC
				**Linear**				
$H(HCPO)_1H$	*C*s	1.73	0.23	259	1.34	0.57	545	68:32
$H(HCPO)_2H$	*C*s	1.74	0.27	265	1.38	0.56	484	65:35
$H(HCPO)_3H$	*C*s	1.70	0.35	316	1.46	0.47	383	55:45
$H(HCPO)_4H$	*C*s	1.70	0.36	319	1.48	0.46	371	54:46
$H(HCPO)_5H$	*C*s	1.68	0.38	334	1.50	0.43	345	51:49
$H(HCPO)_6H$	*C*s	1.69	0.39	337	1.51	0.43	336	50:50
$H(HCPO)_7H$	*C*s	1.68	0.40	346	1.52	0.41	321	48:52
$H(HCPO)_8H$	*C*s	1.68	0.41	348	1.57	0.34	263	43:57
				**Cyclic**				
$(HCPO)_2$	D_{2h}	1.67	0.46	322	1.57	0.34	263	45:55
**$(HCPO)_3$**	**D_{3h}**	**1.70**	**0.46**	**336**	**1.48**	**0.44**	**284**	**46:54**
$(HCPS)_3$	D_{3h}	1.61	0.57	335	1.41	0.53	398	54:46
$(HCPNH)_3$[a]	C_{3h}	1.59	0.54	463	1.52	0.42	291	39:61
$(HCPF_2)_3$	D_{3h}				1.65	0.18	86	
$(HCPH_2)_3$[a]	*C*s	1.08	0.76	3000	1.71	0.13	88	3:97
$(HCNO)_3$	D_{3h}	1.62	0.63	461	1.09	0.91	2837	86:14
$(HCSF)_3$[a]	*C*s	1.59	0.51	1003	1.56	0.36	210	17:83
$(NPO)_3$	D_{3h}	1.66	0.43	352	1.62	0.31	210	37:63
$(NNO)_3$	D_{3h}	1.42	0.78	1226	1.38	0.62	683	36:64
$(H_2BPO)_3$	C_{3v}				1.88	0.08	67	
$(H_2SiPO)_3$	C_{3v}				1.61	0.36	34	
$(MeCPO)_3$[a]	C_{3h}	1.72	0.46	313	1.44	0.48	307	50:50
$(FCPO)_3$	D_{3h}	1.71	0.51	326	1.42	0.52	281	46:54

[a] Due to the lack of symmetry, we present the average values.

Table S3. NRT bond orders (total, covalent and ionic), and sum of bond orders (total valence, covalent and ionic components, and total electron count).

		Total		Covalent		Ionic		X				Y				Z			
	Symm	YZ	XY	YZ	XY	YZ	XY	Val	Cov	Ion	e^-	Val	Cov	Ion	e-	Val	Cov	Ion	e^-
								**Linear**											
$H(HCPO)_1H$	C_s	1.49	1.51	0.69	1.16	0.80	0.35	3.51	2.65	0.86	8.00	4.00	2.82	1.18	8.00	1.49	0.69	0.80	8.00
$H(HCPO)_2H$	C_s	1.50	1.50	0.69	1.12	0.81	0.38	3.50	2.62	0.88	8.00	4.00	2.50	1.50	8.00	1.50	0.69	0.81	8.00
$H(HCPO)_3H$	C_s	1.61	1.39	0.74	0.94	0.87	0.45	3.39	2.35	1.05	8.00	4.00	2.38	1.62	8.00	1.61	0.74	0.87	8.00
$H(HCPO)_4H$	C_s	1.62	1.38	0.74	0.92	0.88	0.46	3.38	2.33	1.05	8.00	4.00	2.36	1.64	8.00	1.62	0.74	0.88	8.00
$H(HCPO)_5H$	C_s	1.65	1.35	0.76	0.88	0.89	0.47	3.35	2.30	1.06	8.00	4.00	2.34	1.66	8.00	1.65	0.76	0.89	8.00
$H(HCPO)_6H$	C_s	1.65	1.35	0.76	0.87	0.90	0.48	3.35	2.29	1.06	8.00	4.00	2.33	1.67	8.00	1.65	0.76	0.90	8.00
$H(HCPO)_7H$	C_s	1.67	1.33	0.77	0.85	0.91	0.48	3.33	2.26	1.06	8.00	4.00	2.32	1.68	8.00	1.67	0.77	0.91	8.00
$H(HCPO)_8H$	C_s	1.68	1.32	0.77	0.84	0.91	0.48	3.32	2.26	1.06	8.00	4.00	2.31	1.69	8.00	1.68	0.77	0.91	8.00
								**Cyclic**											
$(HCPO)_2$	D_{2h}	1.76	1.04	0.76	0.70	1.00	0.34	3.07	2.08	0.98	7.62	3.81	2.15	1.67	8.00	1.75	0.76	0.99	8.00
**$(HCPO)_3$**	**D_{3h}**	**1.63**	**1.12**	**0.71**	**0.73**	**0.91**	**0.38**	**3.23**	**2.14**	**1.09**	**7.72**	**3.86**	**2.18**	**1.68**	**8.00**	**1.63**	**0.71**	**0.91**	**8.00**
$(HCPS)_3$	D_{3h}	1.64	1.09	1.22	0.78	0.42	0.31	3.31	2.39	0.92	7.86	3.83	2.79	1.04	8.00	1.64	1.22	0.42	7.89
$(HCPNH)_3$[a]	C_{3h}	1.59	1.11	0.88	0.75	0.70	0.36	3.25	2.22	1.02	7.73	3.80	2.38	1.42	7.96	2.56	1.48	1.08	7.90
$(HCPF_2)_3$	D_{3h}	0.75	1.19	0.25	0.72	0.50	0.47	3.37	2.12	1.25	7.73	3.88	1.94	1.94	8.00	0.75	0.25	0.50	8.00
$(HCPH_2)_3$[a]	C_s	0.83	1.11	0.81	0.80	0.01	0.31	3.19	2.30	0.89	7.81	3.88	3.24	0.64	7.96	0.83	0.82	0.01	1.97
$(HCNO)_3$	D_{3h}	1.33	1.21	1.15	0.93	0.18	0.28	3.67	2.69	0.98	7.99	3.75	3.00	0.75	8.00	1.63	1.26	0.37	8.00
$(HCSF)_3$[a]	C_s	0.58	1.17	0.25	0.96	0.33	0.22	3.33	2.61	0.72	7.78	2.90	2.12	0.78	8.00	0.60	0.23	0.37	8.00
$(NPO)_3$	D_{3h}	1.74	1.02	0.74	0.51	1.00	0.51	2.12	1.02	1.10	7.88	3.77	1.76	2.01	7.88	1.74	0.74	1.00	7.87
$(NNO)_3$	D_{3h}	1.59	1.07	1.37	0.93	0.23	0.15	2.55	2.16	0.39	8.00	3.58	3.12	0.47	8.00	1.87	1.60	0.26	8.00
$(H_2BPO)_3$	C_{3v}	2.02	0.94	0.91	0.86	1.11	0.08	3.73	3.37	0.36	7.87	3.90	2.62	1.27	7.90	2.02	0.91	1.11	7.98
$(H_2SiPO)_3$	C_{3v}	1.64	1.14	0.75	0.79	0.89	0.35	3.25	2.46	0.80	7.81	3.91	2.32	1.59	7.97	1.64	0.75	0.89	8.00
$(MeCPO)_3$[a]	C_{3h}	1.60	1.14	0.69	0.76	0.90	0.39	3.26	2.41	0.85	7.71	3.85	2.19	1.66	8.00	1.60	0.69	0.90	8.00
$(FCPO)_3$	D_{3h}	1.62	1.11	0.74	0.77	0.88	0.34	3.23	2.17	1.06	7.70	3.85	2.29	1.57	8.00	1.62	0.74	0.88	8.00
$(H_2BF)_3$	C_{3v}		1.00		0.24		0.76	3.98	2.29	1.69	7.98	2.01	0.49	1.52	7.98				
$(HCCl)_3$	C_{3v}		0.99		0.65		0.35	2.99	2.12	0.87	7.97	1.99	1.30	0.69	8.00				
$(HCBr)_3$	C_{3v}		1.00		0.69		0.31	2.99	2.20	0.80	7.99	1.99	1.37	0.62	7.99				
$(HCI)_3$	C_{3v}		0.96		0.80		0.16	2.92	2.41	0.51	7.91	1.92	1.59	0.33	7.93				
$(NF)_3$	C_{3v}		1.00		0.39		0.61	2.00	0.79	1.21	8.00	2.00	0.79	1.21	8.00				
$(NCl)_3$	C_{3v}		1.00		0.80		0.20	2.01	1.61	0.40	8.00	1.99	1.59	0.40	8.00				
$(NBr)_3$	C_{3v}		0.99		0.85		0.13	2.01	1.72	0.28	8.00	2.00	1.72	0.28	8.00				
$(NI)_3$	C_{3v}		1.01		0.83		0.18	2.01	1.66	0.35	8.00	2.00	1.66	0.35	8.00				

[a] Due to the lack of symmetry, we present the average values.

Stability Analysis

To test the stability of the described systems, we started by studying the energetics of the oligomerization of the linear and cyclic molecules by releasing H_2, according to:

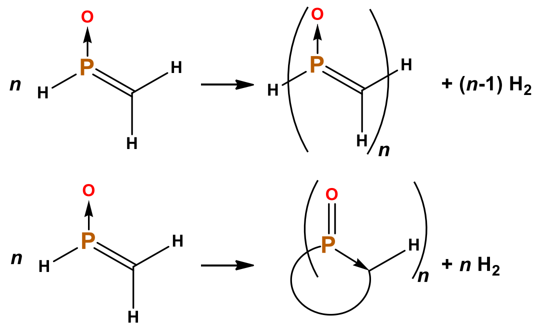

While the enthalpy of the linear oligomerization has a clear lowering trend (brown circles in Fig. S2), the Gibbs energy (blue circles) is hindered by a negative entropy that would only permit long chains (unless the H_2 is vented from the solution). The square cycle with n = 2 would be both endergonic and endothermic, but the six-membered ring with n = 3 is highly stable (ΔH_r = -139 kJ.mol^{-1}, ΔG_r = -129 kJ.mol^{-1}, see squares in Fig. S2), which makes it, in principle, a good candidate for synthesis. For n > 3 we observed that other configurations with intracyclic bond formation are energetically preferred (see below the optimal XYZ geometries), and therefore we did not extend the analysis beyond the six-membered ring.

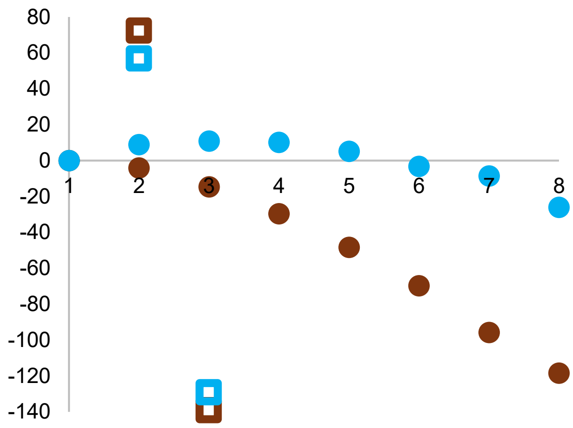

Figure S2. Enthalpy (in brown) and Gibbs energy (in light blue) of formation of the linear (filled circles) and cyclic (empty squares) methylenephosphine oxide oligomers, as a function of the monomer units, with H_2 liberation in standard conditions.

A final stability test was the estimation of the strength of the P-C bond in $(HCPO)_3$. The force constant,[1,2] which estimates the "stiffness" of the interaction, gave a value of 507 N.m^{-1}, corresponding to a regular covalent bond (for reference, the standard P-C covalent bond in methylphosphine has a slightly higher value of k_e = 671 N.m^{-1}, while the strong charge transfer interaction of ammonia-borane has k_e = 188 N.m^{-1}). We also carried out an intrinsic bond strength index (IBSI) analysis as appears in the IGMPlot package,[3,4] which gives a value of 0.71, corresponding to a stable covalent bond (the P-C value in $MePH_2$ is 0.49, indicating that for this method the bond is weaker). An attempt to break the P-C bond provided an open-shell singlet intermediate with a relative energy greater than 200 kJ.mol^{-1}, providing another evidence of the stability of the ring.

[1] J. Grunenberg, *Inorg. Chem.* **2022**, *61*, 20–22.
[2] A. Blahman, S. Kozuch, *Isr. J. Chem.* **2023**, *63*, e202200072.
[3] J. Klein, H. Khartabil, J.-C. Boisson, J. Contreras-García, J.-P. Piquemal, E. Hénon, *J. Phys. Chem. A* **2020**, *124*, 1850–1860.
[4] C. Lefebvre, J. Klein, H. Khartabil, J.-C. Boisson, E. Hénon, *J. Comput. Chem.* **2023**, *44*, 1750–1766.

XYZ Geometries:

Azaborole
```
C -0.051523 -3.425898  0.000000
C -1.425915 -3.206103  0.000000
C -1.941990 -1.913906  0.000000
C -1.086075 -0.822589  0.000000
C  0.290977 -1.068585  0.000000
C  0.824064 -2.350869  0.000000
H -2.099080 -4.054293  0.000000
H -3.014959 -1.764962  0.000000
B -1.378538  0.767088  0.000000
F -1.941990  1.291717  1.140613
H  1.894501 -2.518055  0.000000
H  0.333884 -4.436966  0.000000
N  0.217212  1.242980  0.000000
C  0.657471  2.494622  0.000000
C  2.011963  2.766372  0.000000
C  2.898314  1.694572  0.000000
C  2.422445  0.394589  0.000000
C  1.049292  0.189086  0.000000
H -0.104480  3.263736  0.000000
H  2.361664  3.787911  0.000000
H  3.965248  1.877083  0.000000
H  3.097119 -0.449408  0.000000
F -1.941990  1.291717 -1.140613
```

BODIPY
```
C  0.000000  1.209151  1.104067
N  0.000000  1.245717 -0.280156
B  0.000000  0.000000 -1.233294
F  1.136636  0.000000 -2.007568
N  0.000000 -1.245717 -0.280156
H  0.000000  0.000000  2.861075
C  0.000000 -1.209151  1.104067
C  0.000000  0.000000  1.778352
C  0.000000  2.518881 -0.656428
C  0.000000 -2.518881 -0.656428
H  0.000000 -2.780033 -1.703794
C  0.000000 -2.539859  1.578309
C  0.000000  2.539859  1.578309
C  0.000000  3.363129  0.471192
C  0.000000 -3.363129  0.471192
H  0.000000  2.780033 -1.703794
F -1.136636  0.000000 -2.007568
H  0.000000 -2.828660  2.617162
H  0.000000  2.828660  2.617162
H  0.000000  4.439526  0.450635
H  0.000000 -4.439526  0.450635
```

Linear 8
```
P  0.037764 -1.968306  0.000000
O -0.811077 -3.147632  0.000000
C  1.698187 -1.904149  0.000000
C -0.658885 -0.402679  0.000000
H  0.000000  0.454355  0.000000
C -4.160603  3.931017  0.000000
P -5.537414  4.852977  0.000000
P -4.171773  2.212011  0.000000
O -6.926592  4.434190  0.000000
C -5.190762  6.548245  0.000000
C -2.635769  1.578372  0.000000
P -6.341420  7.724340  0.000000
O -7.790245  7.682038  0.000000
O -5.437549  1.501619  0.000000
H -3.213517  4.452842  0.000000
H -4.165498  6.897081  0.000000
H -5.738811  8.986388  0.000000
H -1.810778  2.277072  0.000000
P -2.294218 -0.101643  0.000000
O -3.378411 -1.067766  0.000000
P  2.734980 -3.272076  0.000000
O  2.168026 -4.611299  0.000000
C  4.332147 -2.827663  0.000000
P  5.673238 -3.910288  0.000000
O  5.423521 -5.345407  0.000000
C  7.108523 -3.096670  0.000000
P  8.695630 -3.801622  0.000000
O  8.802267 -5.263760  0.000000
C  9.888685 -2.693869  0.000000
H  2.148085 -0.921195  0.000000
H  4.535557 -1.765894  0.000000
H  7.041615 -2.017213  0.000000
H  9.640752 -1.644042  0.000000
H  10.922150 -3.001791  0.000000
```

Linear 8 Cis
```
C -3.544153  4.675847  0.000000
P -5.157106  4.319465  0.000000
P -2.277451  3.511644  0.000000
O -5.723415  2.978056  0.000000
C -6.279311  5.629733  0.000000
C -2.713476  1.919379  0.000000
P -5.930728  7.234304  0.000000
O -6.826689  8.374753  0.000000
O -0.916148  4.037544  0.000000
H -3.207626  5.703502  0.000000
H -7.323339  5.333158  0.000000
H -4.559348  7.501723  0.000000
H -3.769161  1.672685  0.000000
P -1.611200  0.603734  0.000000
O -2.178367 -0.741111  0.000000
C  0.000000  0.974607  0.000000
H  0.286723  2.019913  0.000000
P  1.274115 -0.173525  0.000000
O  2.638608  0.349073  0.000000
C  0.849314 -1.770090  0.000000
P  1.950251 -3.089354  0.000000
O  1.367076 -4.430320  0.000000
C  3.558477 -2.725604  0.000000
P  4.848336 -3.872958  0.000000
O  6.200566 -3.307526  0.000000
C  4.439741 -5.462529  0.000000
P  5.554721 -6.804411  0.000000
O  4.911998 -8.127465  0.000000
C  7.138298 -6.440672  0.000000
H -0.206265 -2.016239  0.000000
H  3.837911 -1.678616  0.000000
H  3.383417 -5.703073  0.000000
H  7.439132 -5.403509  0.000000
H  7.872121 -7.231092  0.000000
```

Linear 7
```
P -1.224626  0.023730  0.000000
O -2.659212  0.253490  0.000000
C  0.000000  1.143304  0.000000
C -0.620091 -1.584550  0.000000
C -0.649955 -4.386859  0.000000
P -1.316754 -5.902349  0.000000
P -1.579723 -2.938057  0.000000
O -2.712357 -6.300401  0.000000
C -0.111992 -7.146344  0.000000
P -0.446724 -8.756582  0.000000
O -1.688551 -9.504793  0.000000
O -3.029190 -3.022749  0.000000
H  0.429391 -4.321483  0.000000
H  0.939850 -6.888475  0.000000
H  0.741826 -9.493668  0.000000
H  0.450076 -1.738080  0.000000
H  1.011362  0.761395  0.000000
P -0.233598  2.846694  0.000000
O -1.584655  3.384472  0.000000
C  1.208224  3.662054  0.000000
P  1.380961  5.378765  0.000000
O  0.180715  6.203978  0.000000
C  2.967360  5.829143  0.000000
P  3.574796  7.457140  0.000000
O  2.602271  8.553904  0.000000
C  5.200750  7.534182  0.000000
H  5.703825  8.488034  0.000000
H  2.104154  3.057016  0.000000
H  3.689512  5.024029  0.000000
H  5.777077  6.622330  0.000000
```

Linear 6
```
P -0.810911 -1.222216  0.000000
O -2.264695 -1.247588  0.000000
C  0.259537 -2.483770  0.000000
C  0.000000  0.298528  0.000000
C  0.361085  3.074681  0.000000
P -0.079080  4.668554  0.000000
P -0.764204  1.767591  0.000000
O -1.399125  5.272618  0.000000
C  1.297020  5.723152  0.000000
P  1.208965  7.364534  0.000000
O  0.097035  8.295530  0.000000
O -2.186324  2.063865  0.000000
H  1.420399  2.857518  0.000000
H  2.299078  5.312328  0.000000
H  2.495828  7.912302  0.000000
H  1.081106  0.310169  0.000000
H  1.309205 -2.224767  0.000000
P -0.173506 -4.156514  0.000000
O -1.586582 -4.509205  0.000000
C  1.159424 -5.125889  0.000000
P  1.166546 -6.864851  0.000000
O -0.126363 -7.555128  0.000000
C  2.666122 -7.496871  0.000000
H  2.810660 -8.565573  0.000000
H  2.115002 -4.619311  0.000000
H  3.520874 -6.838852  0.000000
```

Linear 5
```
P  0.000000  0.637829  0.000000
O -0.487737  2.007375  0.000000
C -0.835830 -0.786643  0.000000
C  1.709155  0.373900  0.000000
P  2.833297  1.583063  0.000000
O  2.666637  3.025925  0.000000
C  4.454040  0.960091  0.000000
P  5.809568  1.887543  0.000000
O  6.032310  3.321211  0.000000
H  2.084848 -0.640099  0.000000
H  4.626543 -0.109078  0.000000
H  6.945326  1.071396  0.000000
H -0.245575 -1.692522  0.000000
P -2.562039 -0.920392  0.000000
O -3.345296  0.307276  0.000000
C -3.045968 -2.494198  0.000000
H -2.257282 -3.234303  0.000000
P -4.692586 -3.058981  0.000000
O -5.756809 -2.051678  0.000000
C -4.808962 -4.681591  0.000000
H -5.774180 -5.162588  0.000000
H -3.910731 -5.278961  0.000000
```

Linear 4
```
P  0.931439 -0.845129  0.000000
O  0.203728 -2.106260  0.000000
C  2.545828 -0.531539  0.000000
C  0.000000  0.620293  0.000000
P -1.646440  0.682835  0.000000
O -2.643922 -0.374589  0.000000
C -2.227274  2.323016  0.000000
P -3.808979  2.759212  0.000000
O -5.052666  2.010195  0.000000
H  0.517000  1.570109  0.000000
H -1.525898  3.148236  0.000000
H -3.916286  4.153814  0.000000
H  2.827844  0.512653  0.000000
P  3.816332 -1.725047  0.000000
O  3.416683 -3.134550  0.000000
C  5.303306 -1.067375  0.000000
H  5.409126  0.006183  0.000000
H  6.181191 -1.693800  0.000000
```

Linear 4 Cis
```
C  0.000000  0.946361  0.000000
P -1.575326  0.484221  0.000000
P  1.377495 -0.118169  0.000000
O -2.092095 -0.879471  0.000000
C -2.784476  1.733013  0.000000
C  1.070770 -1.723849  0.000000
P -2.535961  3.351055  0.000000
O -3.493162  4.445098  0.000000
O  2.667921  0.573863  0.000000
H  0.249074  1.998372  0.000000
H -3.807255  1.371942  0.000000
H -1.183160  3.699495  0.000000
H  0.031918 -2.032008  0.000000
P  2.273460 -2.999589  0.000000
O  1.703642 -4.352842  0.000000
C  3.823670 -2.518948  0.000000
H  4.048103 -1.462478  0.000000
H  4.616089 -3.250755  0.000000
```

Linear 3

P	0.000000	0.300146	0.000000
O	1.194263	1.132840	0.000000
C	-0.201944	-1.328890	0.000000
C	-1.532229	1.136242	0.000000
P	1.085965	-2.509360	0.000000
C	0.538602	-4.039240	0.000000
P	-1.723291	2.763450	0.000000
O	-0.801565	3.886995	0.000000
O	2.457304	-1.994959	0.000000
H	-1.224669	-1.681275	0.000000
H	-2.457313	0.573037	0.000000
H	-3.086252	3.078151	0.000000
H	-0.524765	-4.221009	0.000000
H	1.226299	-4.870112	0.000000

Linear 2

P	1.201746	-0.352448	0.000000
O	0.689001	-1.723985	0.000000
C	2.721960	0.216572	0.000000
C	0.000000	0.924085	0.000000
H	-2.246653	1.978443	0.000000
P	-1.622126	0.726652	0.000000
H	2.884151	1.283265	0.000000
O	-2.507865	-0.428682	0.000000
H	0.322003	1.958442	0.000000
H	3.565354	-0.455816	0.000000

Linear 1

P	0.000000	0.282750	0.000000
O	-1.445551	0.046566	0.000000
C	1.283527	-0.703485	0.000000
H	2.257480	-0.235341	0.000000
H	0.403248	1.621693	0.000000
H	1.202519	-1.779214	0.000000

$(HCPO)_2$

C	0.000000	1.250729	0.000000
C	0.000000	-1.250729	0.000000
P	0.000000	0.000000	1.150252
P	0.000000	0.000000	-1.150252
O	0.000000	0.000000	2.601440
O	0.000000	0.000000	-2.601440
H	0.000000	2.322407	0.000000
H	0.000000	-2.322407	0.000000

$(HCPO)_3$

C	0.000000	1.642987	0.000000
C	1.422869	-0.821494	0.000000
C	-1.422869	-0.821494	0.000000
P	1.486087	0.857993	0.000000
P	0.000000	-1.715986	0.000000
P	-1.486087	0.857993	0.000000
O	2.748962	1.587114	0.000000
O	0.000000	-3.174228	0.000000
O	-2.748962	1.587114	0.000000
H	0.000000	2.725043	0.000000
H	2.359956	-1.362521	0.000000
H	-2.359956	-1.362521	0.000000

$(MeCPO)_3$

C	0.000000	-1.675189	0.000000
C	-1.450756	0.837595	0.000000
C	1.450756	0.837595	0.000000
P	-1.469002	-0.852723	0.000000
P	-0.003979	1.698554	0.000000
P	1.472981	-0.845831	0.000000
O	-2.746406	-1.569272	0.000000
O	0.014174	3.163094	0.000000
O	2.732233	-1.593822	0.000000
C	0.015961	-3.193883	0.000000
C	-2.773964	1.583119	0.000000
C	2.758003	1.610764	0.000000
H	-2.589421	2.655719	0.000000
H	-3.361166	1.327479	0.880785
H	-3.361166	1.327479	-0.880785
H	-1.005209	-3.570364	0.000000
H	0.530952	-3.574595	-0.880785
H	0.530952	-3.574595	0.880785
H	3.594631	0.914645	0.000000
H	2.830214	2.247116	0.880785
H	2.830214	2.247116	-0.880785

$(FCPO)_3$

C	0.000000	1.641595	0.000000
C	1.421663	-0.820797	0.000000
C	-1.421663	-0.820797	0.000000
P	1.520294	0.877742	0.000000
P	0.000000	-1.755485	0.000000
P	-1.520294	0.877742	0.000000
O	2.780656	1.605413	0.000000
O	0.000000	-3.210825	0.000000
O	-2.780656	1.605413	0.000000
F	0.000000	2.984354	0.000000
F	2.584526	-1.492177	0.000000
F	-2.584526	-1.492177	0.000000

$(HCPS)_3$

C	0.000000	1.638372	0.000000
C	1.418872	-0.819186	0.000000
C	-1.418872	-0.819186	0.000000
P	1.506253	0.869635	0.000000
P	0.000000	-1.739271	0.000000
P	-1.506253	0.869635	0.000000
S	3.149782	1.818527	0.000000
S	0.000000	-3.637055	0.000000
S	-3.149782	1.818527	0.000000
H	0.000000	2.719876	0.000000
H	2.355482	-1.359938	0.000000
H	-2.355482	-1.359938	0.000000

$(HCPNH)_3$

C	0.000000	1.646350	0.000000
C	1.425781	-0.823175	0.000000
C	-1.425781	-0.823175	0.000000
P	1.491696	0.859503	0.000000
P	-0.001497	-1.721598	0.000000
P	-1.490199	0.862095	0.000000
N	2.745722	1.742155	0.000000
N	0.135890	-3.248943	0.000000
N	-2.881612	1.506787	0.000000
H	0.021287	2.727267	0.000000
H	2.351239	-1.382069	0.000000
H	-2.372526	-1.345198	0.000000
H	3.644127	1.275945	0.000000
H	-2.927064	2.517935	0.000000
H	-0.717063	-3.793879	0.000000

$(HCPF_2)_3$

C	0.000000	1.645793	0.000000
P	1.469118	0.848196	0.000000
P	-1.469118	0.848196	0.000000
F	2.385005	1.376983	1.142589
F	-2.385005	1.376983	-1.142589
C	1.425299	-0.822896	0.000000
C	-1.425299	-0.822896	0.000000
P	0.000000	-1.696392	0.000000
F	0.000000	-2.753966	-1.142589
F	0.000000	-2.753966	1.142589
F	-2.385005	1.376983	1.142589
F	2.385005	1.376983	-1.142589
H	0.000000	2.728510	0.000000
H	2.362959	-1.364255	0.000000
H	-2.362959	-1.364255	0.000000

$(HCPF_2)_3$ Isomer

C	0.018781	1.496559	0.000000
P	-0.471027	0.681524	1.599784
P	-0.471027	0.681524	-1.599784
F	0.535227	1.505125	2.536087
F	-0.595753	2.743393	0.000000
C	0.603938	-0.822254	1.376155
C	0.603938	-0.822254	-1.376155
P	0.113355	-1.959954	0.000000
F	0.535227	-1.537131	-2.563684
F	-1.470298	-1.656433	0.000000
F	0.535227	1.505125	-2.536087
F	0.535227	-1.537131	2.563684
H	1.100260	1.675850	0.000000
H	1.648277	-0.520559	1.236608
H	1.648277	-0.520559	-1.236608

$(HCPH_2)_3$

C	1.483893	-0.759448	-0.087536
P	0.086145	-1.718429	0.092706
P	1.461507	0.940145	-0.045536
H	0.125891	-2.486832	1.280278
H	2.182807	1.423447	-1.161255
C	-1.397554	-0.901401	-0.100571
C	-0.082018	1.630892	0.156081
P	-1.549750	0.790609	-0.035990
H	-2.329838	1.197475	-1.142407
H	-2.409514	1.258388	0.981015
H	2.280295	1.495227	0.960742
H	0.133969	-2.784725	-0.824421
H	2.424474	-1.260918	-0.263834
H	-2.272320	-1.490651	-0.333831
H	-0.130213	2.643463	0.528170

$(HCNO)_3$

C	0.000000	1.348478	0.000000
C	1.167816	-0.674239	0.000000
C	-1.167816	-0.674239	0.000000
N	1.170710	0.675910	0.000000
N	0.000000	-1.351820	0.000000
N	-1.170710	0.675910	0.000000
O	2.258138	1.303737	0.000000
O	0.000000	-2.607473	0.000000
O	-2.258138	1.303737	0.000000
H	0.000000	2.423144	0.000000
H	2.098505	-1.211572	0.000000
H	-2.098505	-1.211572	0.000000

$(HCSF)_3$

C	-0.016554	-0.853111	1.342982
C	-0.016554	-0.853111	-1.342982
C	-0.518816	1.395501	0.000000
S	-0.362170	-1.760463	0.000000
S	-0.016554	0.808968	-1.475454
S	-0.016554	0.808968	1.475454
F	-2.032954	-1.832433	0.000000
F	1.594979	1.167151	-1.555327
F	1.594979	1.167151	1.555327
H	0.032352	-1.398065	2.271822
H	0.032352	-1.398065	-2.271822
H	-0.841753	2.424058	0.000000

$(NPO)_3$

N	0.000000	1.555922	0.000000
N	1.347468	-0.777961	0.000000
N	-1.347468	-0.777961	0.000000
P	1.388896	0.801879	0.000000
P	0.000000	-1.603759	0.000000
P	-1.388896	0.801879	0.000000
O	2.637278	1.522633	0.000000
O	0.000000	-3.045266	0.000000
O	-2.637278	1.522633	0.000000

$(NNO)_3$

N	-1.190049	0.687075	0.000000
N	1.190049	0.687075	0.000000
N	0.000000	-1.374150	0.000000
N	0.000000	1.292290	0.000000
N	1.119156	-0.646145	0.000000
N	-1.119156	-0.646145	0.000000
O	0.000000	2.488185	0.000000
O	2.154831	-1.244092	0.000000
O	-2.154831	-1.244092	0.000000

$(H_2BPO)_3$

B	1.644926	0.949698	0.439074
B	-1.644926	0.949698	0.439074
B	0.000000	-1.899397	0.439074
P	0.000000	1.800492	0.010582
P	-1.559272	-0.900246	0.010582
P	1.559272	-0.900246	0.010582
O	0.000000	3.168343	-0.506644
O	-2.743865	-1.584171	-0.506644
O	2.743865	-1.584171	-0.506644
H	0.000000	-3.021001	0.053862
H	0.000000	-1.730187	1.645192
H	-2.616264	1.510500	0.053862
H	-1.498386	0.865094	1.645192
H	2.616264	1.510500	0.053862
H	1.498386	0.865094	1.645192

$(HSiPO)_3$

Si	1.949398	1.125486	0.601916
Si	-1.949398	1.125486	0.601916
Si	0.000000	-2.250971	0.601916
P	0.000000	1.916471	-0.155863
P	-1.659712	-0.958235	-0.155863
P	1.659712	-0.958235	-0.155863
O	0.000000	3.286306	-0.701873
O	-2.846024	-1.643153	-0.701873
O	2.846024	-1.643153	-0.701873
H	2.841018	1.640263	-0.473892
H	-2.841018	1.640263	-0.473892
H	0.000000	-3.280525	-0.473892

$(H_2BF)_3$

B	0.000000	1.530369	0.234307
B	-1.325339	-0.765185	0.234307
B	1.325339	-0.765185	0.234307
F	-1.161141	0.670385	-0.242434
F	0.000000	-1.340770	-0.242434
F	1.161141	0.670385	-0.242434
H	2.184321	-1.261119	-0.409941
H	1.323003	-0.763836	1.420310
H	-2.184321	-1.261119	-0.409941
H	-1.323003	-0.763836	1.420310
H	0.000000	2.522237	-0.409941
H	0.000000	1.527673	1.420310

$(HCCl)_3$

C	-1.546427	0.892830	0.346717
C	1.546427	0.892830	0.346717
C	0.000000	-1.785660	0.346717
Cl	0.000000	1.657118	-0.206596
Cl	1.435106	-0.828559	-0.206596
Cl	-1.435106	-0.828559	-0.206596
H	-1.422870	0.821494	1.431834
H	1.422870	0.821494	1.431834
H	0.000000	-1.642989	1.431834

(HCBr)$_3$

C	-1.692279	0.977038	0.467647
C	1.692279	0.977038	0.467647
C	0.000000	-1.954076	0.467647
Br	0.000000	1.821022	-0.124446
Br	1.577051	-0.910511	-0.124446
Br	-1.577051	-0.910511	-0.124446
H	-1.538736	0.888389	1.549742
H	1.538736	0.888389	1.549742
H	0.000000	-1.776779	1.549742

(HCI)$_3$

C	-1.836167	1.060111	0.508322
C	1.836167	1.060111	0.508322
C	0.000000	-2.120222	0.508322
I	0.000000	1.999843	-0.087723
I	1.731915	-0.999922	-0.087723
I	-1.731915	-0.999922	-0.087723
H	-1.747794	1.009089	1.599385
H	1.747794	1.009089	1.599385
H	0.000000	-2.018179	1.599385

(NF)$_3$

N	-1.280609	0.739360	0.344742
F	0.000000	1.292119	-0.268133
F	-1.119008	-0.646059	-0.268133
N	1.280609	0.739360	0.344742
N	0.000000	-1.478720	0.344742
F	1.119008	-0.646059	-0.268133

(NCl)$_3$

N	-1.377567	0.795338	0.477138
Cl	0.000000	1.544951	-0.196468
Cl	-1.337967	-0.772476	-0.196468
N	1.377567	0.795338	0.477138
N	0.000000	-1.590677	0.477138
Cl	1.337967	-0.772476	-0.196468

(NBr)$_3$

N	-1.480413	0.854717	0.615549
Br	0.000000	1.704633	-0.123110
Br	-1.476256	-0.852317	-0.123110
N	1.480413	0.854717	0.615549
N	0.000000	-1.709433	0.615549
Br	1.476256	-0.852317	-0.123110

(NI)$_3$

N	0.279164	1.105939	1.581259
I	-0.808330	1.700774	0.000000
I	0.279164	-0.902611	1.629031
N	0.279164	1.105939	-1.581259
N	1.334536	-1.421057	0.000000
I	0.279164	-0.902611	-1.629031

(HCPO)$_3$ Prismane

C	-0.822999	0.917534	0.798526
P	-0.424316	-0.827067	1.099563
P	0.748057	1.310461	0.000000
O	-0.424316	-1.545502	2.370204
C	1.044108	-0.469661	0.000000
C	-0.822999	0.917534	-0.798526
P	-0.424316	-0.827067	-1.099563
O	-0.424316	-1.545502	-2.370204
O	1.638198	2.463486	0.000000
H	-1.573676	1.520656	1.289995
H	1.950780	-1.058505	0.000000
H	-1.573676	1.520656	-1.289995

(HCPO)$_3$ Ladderane

C	-0.742557	0.839532	1.505196
P	-0.087217	-0.657266	1.554746
P	0.110843	1.298766	0.000000
O	-0.087217	-1.827757	2.420234
C	0.795685	-0.465344	0.000000
C	-0.742557	0.839532	-1.505196
P	-0.087217	-0.657266	-1.554746
O	-0.087217	-1.827757	-2.420234
O	0.934686	2.509507	0.000000
H	-1.429749	1.382385	2.131291
H	1.867927	-0.642549	0.000000
H	-1.429749	1.382385	-2.131291

(HCPO)$_4$

C	-0.554231	-2.023077	1.165081
P	0.618940	-1.168777	0.155225
P	-1.823641	-1.587853	0.214882
O	1.798680	-1.795138	-0.444216
C	0.834652	0.531472	0.837898
C	-0.834652	-0.531472	-0.837898
P	1.823641	1.587853	-0.214882
O	3.253054	1.837788	-0.154488
O	-3.253054	-1.837788	0.154488
H	-0.443588	-2.603834	2.065249
H	0.901453	0.649837	1.914504
H	-0.901453	-0.649837	-1.914504
P	-0.618940	1.168777	-0.155225
O	-1.798680	1.795138	0.444216
C	0.554231	2.023077	-1.165081
H	0.443588	2.603834	-2.065249

(HCPO)$_5$

C	-0.118625	-1.204004	0.802722
P	0.899319	-1.175663	-0.715125
P	-1.923966	-0.857275	0.579196
O	0.339238	-1.780716	-1.926656
C	2.606169	-1.335491	-0.278674
C	-2.290334	-0.437966	-1.105744
P	0.218445	0.582391	1.148535
O	0.842814	1.084696	2.372215
O	-2.821741	-1.636772	1.429708
H	0.081381	-1.971309	1.545050
H	3.227369	-2.201796	-0.123773
H	-2.483275	-1.048933	-1.971568
P	-2.071617	1.167241	-0.851481
O	-2.056191	2.396525	-1.631781
C	-1.549908	0.965893	0.845766
H	-2.026805	1.542382	1.633980
C	1.122464	0.683401	-0.452002
P	2.854019	0.287407	-0.216001
O	3.956293	1.204020	0.012550
H	0.856417	1.445102	-1.181237

(HCPO)$_6$

C	1.263131	0.108049	0.202130
P	-0.116473	-0.508560	1.217109
O	-0.038411	-1.801646	1.900490
H	1.972923	0.820956	0.617322
P	2.105177	-1.202469	-0.821903
O	3.548391	-1.022448	-0.961394
C	1.324939	-2.781428	-0.629178
H	1.435269	-3.554856	0.112248
P	0.295724	-2.526095	-1.881318
O	-0.825154	-3.198109	-2.520963
C	0.865231	-0.858584	-2.182751
P	0.116473	0.508560	-1.217109
O	0.038411	1.801646	-1.900490
C	-1.263131	-0.108049	-0.202130
P	-2.105177	1.202469	0.821903
O	-3.548391	1.022448	0.961394
C	-1.324939	2.781428	0.629178
P	-0.295724	2.526095	1.881318
O	0.825154	3.198109	2.520963
C	-0.865231	0.858584	2.182751
H	1.175986	-0.568295	-3.183129
H	-1.972923	-0.820956	-0.617322
H	-1.435269	3.554856	-0.112248
H	-1.175986	0.568295	3.183129
